# Mood Monitoring Over One Year for People With Chronic Obstructive Pulmonary Disease Using a Mobile Health System: Retrospective Analysis of a Randomized Controlled Trial

**DOI:** 10.2196/14946

**Published:** 2019-11-22

**Authors:** Maxine E Whelan, Carmelo Velardo, Heather Rutter, Lionel Tarassenko, Andrew J Farmer

**Affiliations:** 1 Nuffield Department of Primary Care Health Sciences University of Oxford Oxford United Kingdom; 2 Institute of Biomedical Engineering Department of Engineering Science University of Oxford Oxford United Kingdom

**Keywords:** pulmonary disease, chronic obstructive, self-management, telemedicine, computers, handheld, anxiety, depression

## Abstract

**Background:**

Comorbid anxiety and depression can add to the complexity of managing treatment for people living with chronic obstructive pulmonary disease (COPD). Monitoring mood has the potential to identify individuals who might benefit from additional support and treatment.

**Objective:**

We used data from the sElf-management anD support proGrammE (EDGE) trial to examine: (1) the extent to which the mood-monitoring components of a mobile health system for patients with COPD were used by participants; (2) the levels of anxiety and depression symptoms among study participants; (3) the extent to which videos providing advice about coping with low mood were viewed; and (4) the characteristics of participants with differing levels of mood and utilization of mood monitoring.

**Methods:**

A total of 107 men and women with a clinical diagnosis of COPD, aged ≥40 years old, were recruited to the intervention arm of the EDGE trial. Participants were invited to complete the Patient Health Questionnaire-8 and the Generalized Anxiety Disorder-7 test every four weeks using a tablet computer. Mood disturbance based on these measures was defined as a score ≥5 on either scale. Participants reporting a mood disturbance were automatically directed (signposted) to a stress or mood management video. Study outcomes included measures of health status, respiratory quality of life, and symptoms of anxiety and depression.

**Results:**

Overall, 94 (87.9%) participants completed the 12-month study. A total of 80 participants entered at least one response each month for at least ten months. On average, 16 participants (range 8-38 participants) entered ≥2 responses each month. Of all the participants, 47 (50%) gave responses indicating a mood disturbance. Participants with a mood disturbance score for both scales (n=47) compared with those without (n=20) had lower health status (*P*=.008), lower quality of life (*P*=.009), and greater anxiety (*P*<.001) and increased depression symptoms (*P*<.001). Videos were viewed by 64 (68%) people over 12 months. Of the 220 viewing visualizations, 70 (34.7%) began after being signposted. Participants signposted to the stress management video (100%; IQR 23.3-100%) watched a greater proportion of it compared to those not signposted (38.4%; IQR 16.0-68.1%; *P*=.03), whereas duration of viewing was not significantly different for the mood management video.

**Conclusions:**

Monitoring of anxiety and depression symptoms for people with COPD is feasible. More than half of trial participants reported scores indicating a mood disturbance during the study. Signposting participants to an advisory video when reporting increased symptoms of a mood disturbance resulted in a longer view-time for the stress management video. The opportunity to elicit measures of mood regularly as part of a health monitoring system could contribute to better care for people with COPD.

## Introduction

Comorbid anxiety and depression affect as many as 50% of people with chronic obstructive pulmonary disease (COPD) [1,2] and can add to the complexity of managing treatment [3]. Depression and anxiety have been linked with mortality and hospital readmission, respectively [4]. Digital health studies to date involving people with COPD have focused on monitoring physiological variables, including oxygen saturation and pulse rate, symptoms, and identifying a practical daily measurement regimen to support self-management of their condition [5]. There is scope, however, for people with COPD to monitor not only vital signs and symptoms but also mood. Self-reporting mood can result in a greater awareness of one’s own symptoms, which could result in several outcomes such as preventing deterioration in feelings of anxiety [6]. The use of digital technologies to support remote patient monitoring has become increasingly feasible for a wide range of health conditions due to the use of tablet computers and other readily available devices [7].

Monitoring mood is most often an active process that involves the manual entry of information, including symptoms of anxiety and depression [8]. Many mood-tracking mobile applications are available and could improve mental health and well-being but lack research evidence [9]. Technologies to self-report mood have provided clinicians and patients with a rich understanding of its variation among people with other conditions, and mental health applications are particularly considered a promising tool to extend mental health care beyond the clinic setting [10]. It is anticipated that the act of self-monitoring mood may reduce symptoms of anxiety and depression by increasing emotional self-awareness [11]. Bipolar disorder offers an exemplar where self-monitoring mood is prevalent [12], with self-monitoring mood on a daily basis being described as being of little or no inconvenience in people with bipolar disorder [13]. Digital technologies can be used to report measures of mood responses with ease. To date, there have been a number of such studies with bipolar patients, and findings suggest the time taken to complete mood questions is short [14], with rates of completion ranging from 73.4-100% [14-17].

We are not aware of any published studies involving digital health approaches to self-monitoring of mood in people with COPD. Current nonpharmacological guidelines recommend cognitive behavioral therapy, counselling, and self-help approaches [18]. It is known that untreated comorbid anxiety and depression can have overwhelming consequences on people with COPD [19], such that it impacts their physical, psychological, and social resilience [20]. For instance, depression in patients hospitalized for an acute exacerbation (a severe worsening of COPD symptoms) can adversely affect chances of survival [21,22] and has been associated with longer hospital length of stay, increased symptom burden, and reduced quality of life [23], as well as a reduction in daily step counts [24]. Consequently, monitoring of mood symptoms could be a logical addition to self-management interventions involving people with COPD; however, it is currently unclear how well people with COPD might adhere to self-monitoring mood and how their symptoms of anxiety and depression may vary over time.

The aim of this analysis, carried out on data obtained as part of a randomized trial which involved mood monitoring within a wider mobile health (mHealth) self-management application [25], was to examine: (1) the extent to which the mood monitoring components of a COPD digital health system were used by participants over 12 months; (2) the levels of anxiety and depression symptoms among study participants; (3) the extent to which videos providing advice about coping with low mood were viewed; and (4) the characteristics of participants with differing levels of mood and utilization of mood self-monitoring.

## Methods

### Overview

A digital health system called sElf-management anD support proGrammE (EDGE) was developed and customized for COPD patients [25]. The system supports self-management for patients by using a COPD symptom questionnaire, a Bluetooth-enabled pulse oximeter, and multimedia content. A component of the system included the collection of self-reported mood data by patients.

### Study Population

The data for this study was drawn from 110 participants allocated to the intervention arm of a 12-month randomized controlled trial of the EDGE COPD system. The trial recruited patients with a confirmed diagnosis of COPD, aged ≥40 years old, from primary care and community settings in the Thames Valley (United Kingdom), between 2013 and 2015 [25]. Trial eligibility criteria are reported in the protocol [26]. All participants gave their informed consent to take part. Ethics approval was received from the South Central‚ Berkshire Research Ethics Committee of the UK National Research Ethics Service (Ethics Ref: 12/SC/0437).

### Intervention

Each participant received a low-cost, internet-linked Android tablet computer (Samsung Galaxy Tab II 10.1) with the EDGE mHealth application installed, and an accompanying pulse oximeter (Nonin, Onyx II, 9560BT). All participants had unrestricted access to the tablet computer and pulse-monitoring device for 12 months. Participants were asked to complete a series of standard questions relating to their COPD symptoms daily and a mood questionnaire monthly. The mood questionnaire was completed monthly to reflect clinical practice and even though there are measures to capture daily variations in mood, intensive self-monitoring of mood was not considered necessary in our patient population. After completing the COPD symptom diary, participants were encouraged to wear the pulse oximeter for 30 seconds to record oxygen saturation levels and heart rate.

Participants were asked to use a mood monitoring tool comprising the following mood questions: a 4-item scale which included the Patient Health Questionnaire 2-item measure (PHQ-2) and the General Anxiety Disorder 2-item measure (GAD-2) [27]. These questions were first shown to participants two weeks after they started using the system and then at intervals every four weeks. Participants were prompted to complete the questionnaire via the EDGE system and were offered the opportunity to complete the questionnaire immediately or later. The prompt would remain on the tablet computer until the questionnaire was completed. If the anxiety (GAD-2) or the depression (PHQ-2) measures were scored as >1 point then the relevant full questionnaire (the PHQ-8 or GAD-7) was shown, in line with national guidance [28-30]. PHQ-8 scores of 0-4, 5-9, 10-14, 15-19 and ≥20 were defined as none, mild, moderate, moderate-severe, and severe, respectively. GAD-7 scores of 0-4, 5-9, 10-14 and ≥15 were defined as none, mild, moderate, and severe, respectively. Participants were signposted to videos in the event scores of ≥5 points on either scale were reported by suggesting they might find it helpful to view either the stress or mood management video (or both). Participants were not able to track their mood over time, but if they scored above the ≥5 threshold at any point (or on multiple occasions) they would be signposted each time to the videos. The stress and mood management videos (with durations of 3 minutes 39 seconds and 1 minute 51 seconds, respectively) were also accessible to participants on the tablet computer at any time. A threshold of ≥90% of the duration of the video was applied to define a video as watched to distinguish them from brief video visualizations (<90%). The tablet computer recorded the day and time of entering data, and the day, time, and duration of viewing videos.

### Study Procedures

Research nurses provided participants with brief details on use of the EDGE system and gave out an information booklet outlining how to charge the devices. Participants were informed that the EDGE system was not a replacement for usual care, and that in the event of any deterioration they should contact their general practitioner (GP) or community respiratory nurse. Data was reviewed by a clinician at no less than 4-day intervals, and if either the PHQ-8 or GAD-7 scores were ≥10 then the GP was informed by letter.

### Data Collection

PHQ-8 and GAD-7 scores were recorded via the tablet computer. Age, sex, body mass index (BMI), COPD severity using the Global Initiative for Chronic Obstructive Lung Disease (GOLD) staging [31], Forced Expiratory Volume in 1 second (FEV_1_%), health status using the EuroQol 5-Dimension Questionnaire (EQ-5D) [32], symptoms of anxiety and depression using the Symptom Checklist (SCL10 and SCL20a, respectively) [33], adherence to taking medications using the Medication Adherence Report Schedule (MARS) [34], Beliefs about Medications Questionnaire (BMQ) [35], respiratory quality of life using the St George’s Respiratory Questionnaire for COPD (SGRQ-C) [36], smoking status (current or exsmoker of ≥2 years or exsmoker of <2 years), and number of pack-years were recorded at baseline.

### Data Management

Self-reported mood data was collected via the tablet computer and linked to a study ID. All tablet computers were linked to the Internet by a subscriber identity module (SIM) card. Data was transferred from the EDGE mHealth application to a database held on a secure web server. Anonymized data were extracted for analysis. Participants who withdrew from the study were excluded from analysis.

### Statistical Analysis

Descriptive statistics (frequency, percentage) or mean (SD) or median (IQR) are reported. Comparisons of continuous and categorical variables used a two-tailed independent *t* test and a Chi-square analyses, respectively. The Mann-Whitney U test was completed for continuous variables when data were not normally distributed. The threshold for statistical significance was *P*<0.05 for all comparisons in this exploratory analysis.

Responses to the mood questions were assigned to the 4-week period in which they were completed. Some participants completed the mood questions on more than one occasion during each period of 4 weeks; up to three responses for each period were included, and the response with the greatest score was used for that month. If a participant did not respond to the mood questions for a given 4-week period, then this was recorded as a nonresponse.

Characteristics of participants who provided ≥10 months of responses and participants who provided <10 responses were compared. A second comparison involved participants who recorded a score of ≥5 for both the GAD-7 and PHQ-8 questionnaires on at least one occasion during the 12 months compared to participants who recorded <5 for the tablet-based mood measures throughout the trial.

A video session was defined as either: (1) happening after being signposted; or (2) watched freely with no prompt to watch. Signposted participants were immediately directed to the relevant video(s) where a score of ≥5 was recorded. Video visualizations that happened after a participant was signposted were defined as occurring within 30 minutes of entering the mood responses (within the range of observed response); other video visualizations were defined as being watched without being prompted. Participants signposted to the videos who watched at least 90% of its duration were profiled for their subsequent mood response to describe the short-term impact of this intervention.

## Results

### Summary

Only one of the 107 participants allocated to use the EDGE COPD system did not complete any mood questions. During the 12-month study period, 12 (11.3%) participants withdrew from the trial, of whom five gave the response, “too many things were going on” as a reason for stopping, five died, and two did not give a reason. Of these 12 participants, three withdrew before three months, two withdrew before six months, and seven withdrew before 12 months. In total, 94 participants completed the study. The characteristics of these participants are reported in Table 1.

**Table 1:** Participant characteristics.

Characteristics		Completing study (n=94)
Age (years), mean (SD)		69.7 (9.3)
Male, n (%)		57 (60.6)
**BMI^a^, n (%)**		
	Underweight	3 (5.9)
	Normal weight	14 (27.5)
	Overweight	16 (31.4)
	Obese	18 (35.3)
FEV_1_%^b^, mean (SD)		47.7 (15.9)
**GOLD^c^ staging, n (%)**		
	Moderate	37 (39.4)
	Severe	40 (42.6)
	Very severe	17 (18)
**Smoking status, n (%)**		
	Current smoker	18 (19.2)
	Exsmoker (<2 years)	13 (13.8)
	Exsmoker (≥2 years)	63 (67)
Years smoking, median (IQR)		40 (29-55)
**MRC^d^ dyspnea score, n (%)**		
	2	15 (16)
	3	64 (68.1)
	4	15 (16)
EQ-5D^e^ index, median (IQR)		0.6 (0.5-0.7)
SCL10^f^, median (IQR)		3 (1-9)
SCL20a^f^, median (IQR)		10.5 (7-23)
SGRQ-C^g^, mean (SD)		56.5 (18.8)
BMQ^h^, mean (SD)		32.6 (6.2)
MARS^i^, median (IQR)		24 (23-25)

^a^BMI: body mass index.

^b^FEV_1_%: Forced Expiratory Volume in 1 second.

^c^GOLD: Global Initiative for Chronic Obstructive Pulmonary Disease.

^d^MRC: Medical Research Council.

^e^EQ-5D: EuroQol 5-Dimension Questionnaire.

^f^SCL: Symptom Checklist.

^g^SGRQ-C: St George’s Respiratory Questionnaire for Chronic Obstructive Lung Disease.

^h^BMQ: Beliefs about Medications Questionnaire.

^i^MARS: Medication Adherence Report Schedule.

### Use of the Mood-Monitoring Tool

**Figure 1:**
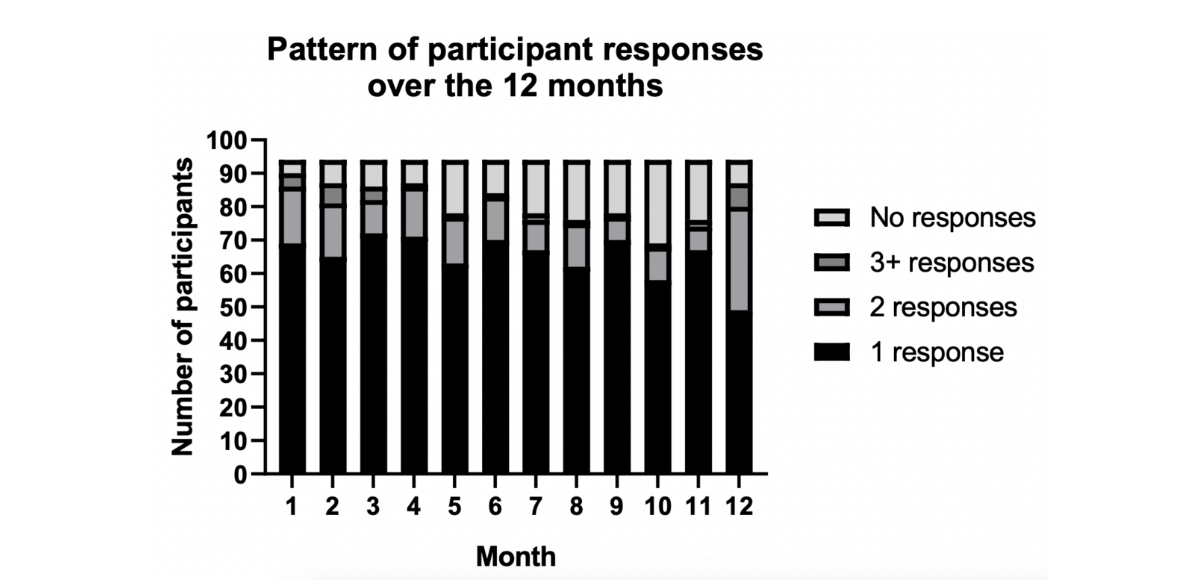
Monthly frequency of responses to the mood questionnaires completed every four weeks.

In total, there were 1200 responses to the mood questions. The distribution of responses is shown in Figure 1. The total amount of missing data was 13.4%. There was a decline in response over time, but 80 (85.1%) participants responded at least once to the mood questionnaire in ≥10 of the 12 months. Smoking status differed between those who used the system in each of the ≥10 months (n=80) compared to those who used the system in <10 months (n=14) (*P*<.001), with no other differences observed (see Multimedia Appendix 1).

### Levels of Mood Disturbance Over the Duration of the Study

**Figure 2:**
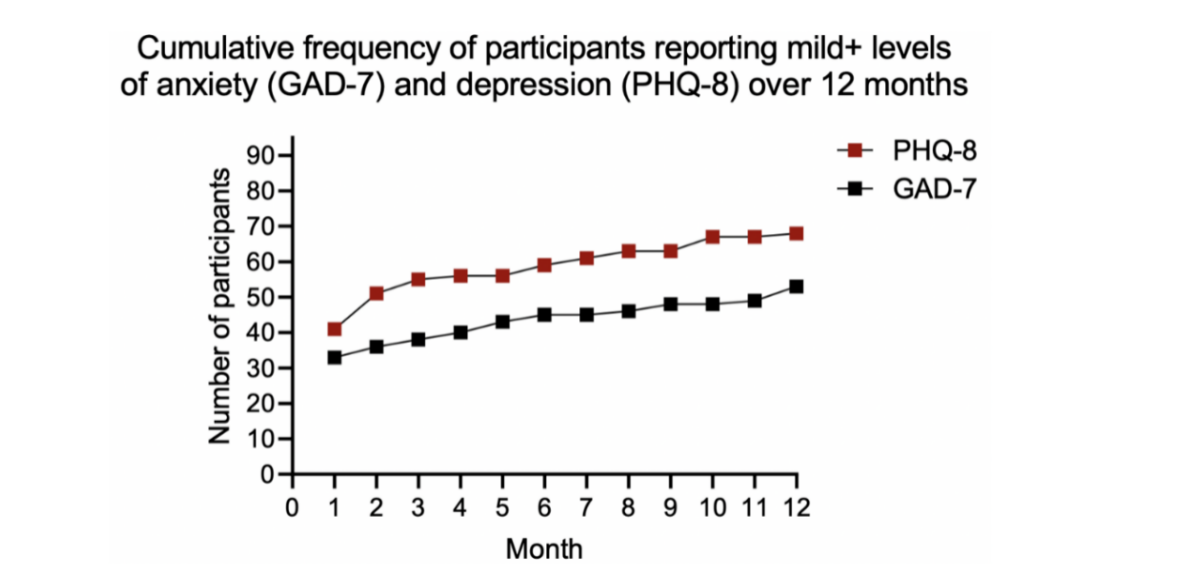
Cumulative frequency of participants with levels of anxiety and depression above their respective clinically defined thresholds. PHQ-8: Patient Health Questionnaire 8-item measure; GAD-7: General Anxiety Disorder 7-item measure.

**Figure 3:**
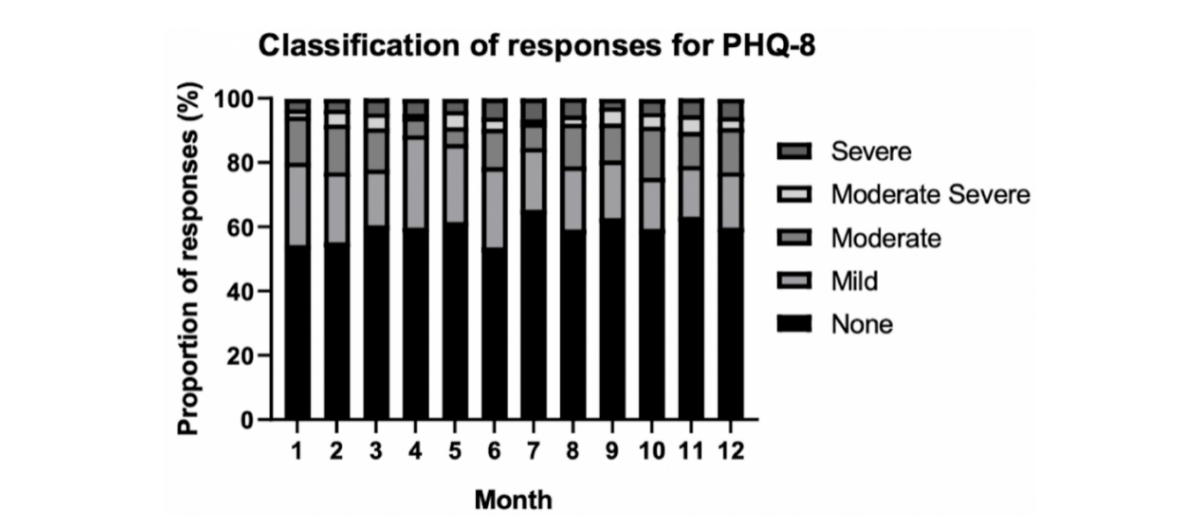
Proportion of, and degree of elevation for, symptoms of depression over the 12 months. PHQ-8: Patient Health Questionnaire 8-item measure.

**Figure 4:**
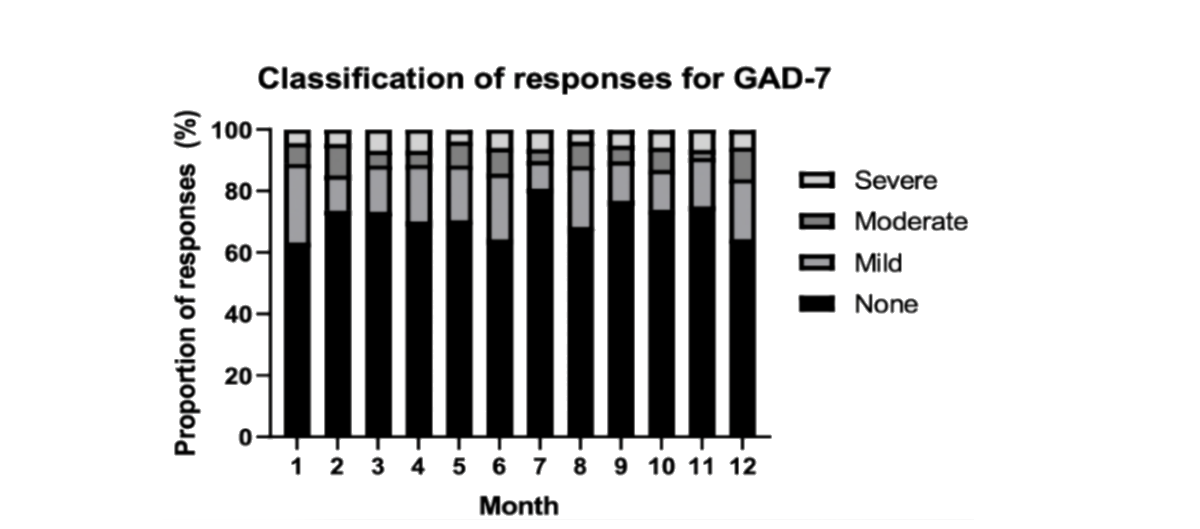
Proportion of, and degree of elevation for, symptoms of anxiety over the 12 months. GAD-7: General Anxiety Disorder 7-item measure.

In the first month of using the system, 41 (43.6%) participants recorded a PHQ-8 score of ≥5, and the total number of participants recording at least one elevated score increased to 68 (72.3%) by 12 months (Figure 2). In comparison, 33 (35.1%) participants reported a GAD-7 score ≥5 in the first month, increasing to 53 (56.4%) participants by 12 months (Figure 2). A detailed breakdown of participant responses for both the PHQ-8 and GAD-7 scores, and their respective thresholds of severity for each of the 12 months, is reported in Figure 3 and Figure 4.

### Comparison of People with Mood Disturbance to Those Without

Overall, 47 (50%) participants had elevated scores for both the PHQ-8 and GAD-7 on at least one occasion and 20 (21.3%) participants recorded no elevated scores on either measure. The remaining participants only reported an elevated score for either PHQ-8 (n=21) or GAD-7 (n=6) during the 12-month monitoring period. Compared to those who reported no changes in the scores, those who responded with an elevated score for both scales during the 12 months (n=47) reported a lower health status (median 0.7 [IQR 0.6-0.8]; versus median 0.5 [IQR 0.4-0.07]; *P*=.008), reduced respiratory quality of life (mean 50.7 [SD 16.8] versus mean 63.3 [SD 17.8]; *P*=.009), greater symptoms of anxiety (median 1 [IQR 0-4] versus median 6 [IQR 2-13]; *P*<.001) and greater symptoms of depression (median 6 [IQR 3-9] versus median 22 [IQR 10-43]; *P*<.001), and no other differences were observed (see Multimedia Appendix 2).

### Accessing and Watching the Mood Management and Stress Videos

In total, 51 (54.3%) participants accessed the mood management video at least once while 41 (43.6%) participants accessed the stress management video. A total of 64 (68.1%) participants watched one of the two videos and 28 (29.8%) participants watched both the mood and the stress management videos during the 12 months. The mood and stress management videos were watched an average of 0.8 (SD 1.0) and 0.9 (SD 1.1) times, respectively. Of the 202 video visualizations logged during the 12 months (an average of 3.2 video visualizations per participant), participants accessed the videos without being prompted a total of 132 times (78 for the mood management video and 54 for the stress video). The median proportion of the duration of the video watched was 49.5% (IQR 12.2-81.1%) for the mood video and 38.4% (IQR 16.0-68.1%) for the stress management videos.

### Proportion Watched by Those Signposted to the Mood Management and Stress Videos

There were a total of 471/1200 responses that used the mood-monitoring tool with a score of ≥5 points for the PHQ-8. In total, 39 video visualizations were watched after being signposted during the 12 months (recorded by 23 participants); 20 (51.3%) of these visualizations involved watching ≥90% of the video duration. The median proportion of the duration of the mood management video watched was 83.7% (IQR 23.2-100.0%). Similarly, 317/1200 responses recorded a score of ≥5 points for GAD-7 and were signposted to the stress management video. In total, 23 participants viewed 31 stress management video visualizations after being signposted during the 12 months and 20 (64.5%) visualizations were viewed for ≥90% of their duration. The median proportion of the stress management video watched was 100% (IQR 23.3-100%). Overall, 8 (14.3%) participants who reported an elevated score for both anxiety and depression during the 12 months watched ≥90% of both videos, while 21 (37.5%) participants did not watch either. In comparison, 4 (17.4%) participants who responded with no elevated scores for symptoms of either anxiety or depression watched at least one of these videos.

### Effect of Signposting Participants Versus Watching the Videos Freely

The proportion of the duration of the stress management video watched was significantly greater for the video visualizations that were signposted (median 100% [IQR 23.3-100.0%]; *P*=.03) compared to those freely watched (median 38.4% [IQR 16.0-68.1%]; *P*=.03) but was not significant for the mood management video. In total, 11 participants accessed the mood and stress management videos upon being signposted for both components at some point during the 12 months.

### The Impact of Signposting on Subsequent Mood Disturbance

Of the participants who were signposted to the mood management or stress video and watched ≥90% of its duration, 12 (54.5%) entered a lower score in the subsequent month, 4 (18.2%) entered a higher score, and the remaining 6 (27.3%) participants entered an identical score. In comparison, of the participants who were signposted but did not watch ≥90% of either video, 9 (52.9%) participants entered a lower score, 4 (23.5%) entered a higher score, and the remaining 2 participants (11.8%) entered an identical score.

## Discussion

### Principal Findings

This study confirms that it is both feasible and acceptable for people with COPD to complete monthly mood questions using a tablet computer–based digital health system. Many participants reported symptoms of anxiety and low mood at least once during the 12 months. We report how this group of participants reported a lower health status and quality of life compared to participants who did not report any symptoms of anxiety or depression. Signposting participants at the point of entering elevated mood responses did result in longer viewing times for the stress-management video but it remains unclear how to optimize the process to make support available to people with COPD. In principle, mood self-monitoring could form an important component of future studies to consider the presence (and severity) of mood disturbance in people with COPD.

To our knowledge, this is the first study to evaluate incorporating longitudinal mood monitoring in digital health systems for people with COPD. Participants in this study were asked to answer a set of mood questions every 4 weeks over 12 months. Electronic self-monitoring of mood has been used extensively in people with bipolar disorder previously [37], with studies lasting 2 weeks [38], to 3 months [39], to 18 months [40]. Participants in the 2-week study were asked to respond to questions at 4 random times per day with no less than 2 hours between each survey [38]. This intense data collection strategy captured mood states and identified triggers in 10 people with bipolar disorder, and the median percentage of completed possible surveys was 78%. In comparison, Scharer et al asked participants to complete daily questions via a mobile application over 18 months and demonstrated its validity in recognizing both manic and depressive episodes [40]. The amount of reported missing data to date has ranged from 6.1-57.9% [37], while in the present analysis 13.4% was missing. This suggests that people with COPD were very compliant in self-monitoring mood over several months. However, the intensity of required reporting in this study was much lower compared with that in the bipolar literature and it is unclear to what extent the amount of missing data would increase if the required reporting was more frequent in people with COPD.

Despite instructions asking participants to complete the questions every four weeks in the current study, many participants responded more frequently. The mode of accessing the mood questions may have in part contributed to its ease of use. Previous studies have used web-based interfaces, personal digital assistants, and smartphones for data collection [37], but the current study used a tablet computer and the mood monitoring element was a part of a multicomponent digital intervention. Participants were also reminded to complete the questions after the 4-week period if they had not, and this reminder persisted on the tablet computer interface until the mood questions were answered. The high adherence rates may also be in part because the mood self-monitoring component formed part of a broader mHealth self-management system which was comprised of daily symptom monitoring and access to other information videos, including inhaler techniques and physical exercises. With a high degree of compliance to use of this element of the EDGE system, the findings support the use of self-monitoring mood in people with COPD even though systematic reviews [41,42] have highlighted that telemonitoring studies more often monitor other symptoms, including dyspnea and sputum [43-45], lung function [46], and physical activity in this patient group [47,48].

Monitoring mood in people with COPD may not yet be common, but the impact of mood disturbance is substantial. A meta-analysis of nearly 40,000 people with COPD highlighted that one quarter experienced clinically significant depressive symptoms (compared with one eighth in controls) [49]. We identified that half of people with COPD experienced clinically significant symptoms of anxiety and depression on at least one occasion over the 12-month period. Not only is the prevalence important but also its influence on self-managing the condition.

Elevated symptoms for anxiety and depression have been associated with several markers of ill health. This includes fatigue and shortness of breath [50,51], poorer health-related quality of life [52-54], frequent hospitalization [55], comorbidities [56], functional limitations, and reduced exercise capacity [57-59]. People with a long-term condition, like COPD, also living with depression or anxiety have a worsened health status compared to people living with depression or anxiety alone [60]. People who reported elevated scores for the PHQ-8 and GAD-7 on at least one occasion over the 12 months had a significantly lower health status, reduced respiratory-related quality of life and elevated symptoms for both anxiety and depression. Symptoms of anxiety and depression are important determinants of health outcomes and health care utilization [61].

Most participants who were signposted to the videos did not watch the video. However, when participants did go on to watch the stress video after being signposted, they watched it for significantly longer. Signposting at the point of someone receiving an elevated score is an important opportunity to deliver relevant information. For instance, in the True Colours longitudinal mood-monitoring study, people with affective disorder were signposted to communicate with their relevant health care team to highlight any concerns with their mood symptoms [62]. In this analysis we focused on how people engaged with the stress management and mood videos, but this could extend to other domains of COPD, including self-reported shortness of breath. In that example, individuals could be directed to breathing technique videos, such as pursed lip breathing techniques, or it could extend even wider to other long-term conditions if people have comorbidities.

### Implications for Future Research and Clinical practice

This analysis demonstrates the current potential of monitoring mood in people with COPD. It appears a feasible and acceptable parameter to monitor over 12 months with a high rate of adherence. Previous work has suggested value in monitoring several factors in people with COPD, including factors that affect activities of daily living, like symptoms [63]. Monitoring mood together with existing parameters of interest (including oxygen saturation and COPD symptoms) may provide a better insight into individual well-being. The findings also suggest that signposting people at the point of reporting an elevated score can lead to longer video viewing times and is a timely, resource-light feature. The integration of signposting to multimedia resources in digital health interventions is therefore encouraged, using health care system–endorsed material where possible, as is the presentation of feedback to allow participants to track their mood scores over time.

### Strengths and Limitations

The delivery of the mood monitoring component within the EDGE system was easy to use and nonobtrusive for participants. It formed part of a wider self-management system which included daily questions around COPD symptoms, demonstrating that the mood questions could be integrated with other components of self-management. As all participants received a reminder if they had an overdue response, we could not comment on the adherence rates if no reminder was sent. We were only able to determine if signposting resulted in an immediate viewing of the video (within 30 minutes) but could not comment on delayed watching habits. Participant insights would have been valuable to decipher why those participants did not follow the signposting.

### Conclusion

Mood monitoring in people with COPD is feasible, with most patients answering the mood questions over 12 months and with some participants entering multiple responses each month. With more than half of participants reporting elevated scores, plus the association this has with health status and quality of life, self-monitoring of mood could be an important, additional component to COPD self-management strategies. In addition, the incorporation of signposting for people with COPD to videos when entering an elevated score demonstrated promise, but further work is needed to optimize this approach.

## Appendix

Multimedia Appendix 1 Overview of statistical comparisons for adherent participants compared with those nonadherent with using the system.

Multimedia Appendix 2 Overview of statistical comparisons for participants with elevated PHQ-8 and GAD-7 scores compared with those with nonelevated scores.

Multimedia Appendix 3 CONSORT-EHEALTH checklist (V 1.6.1).

## Abbreviations

BMI: body mass index
BMQ: beliefs about medications questionnaire
COPD: chronic obstructive pulmonary disorder
FEV1%: forced expiratory volume in 1 second
EDGE: sElf-management anD support proGrammE
EQ-5D: EuroQol 5-Dimension Questionnaire
GAD: General Anxiety Disorder measure
GOLD: Global Initiative for Chronic Obstructive Lung Disease
GP: general practitioner
MARS: Medication Adherence Report Schedule
mHealth: mobile health
NIHR: National Institute for Health Research
PHQ: Patient Health Questionnaire
SCK: Symptom Checklist
SGRQ-C: St George’s Respiratory Questionnaire for Chronic Obstructive Lung Disease questionnaire
SIM: subscriber identity module
