# Supplementary material for: Mood Monitoring Over One Year for People With Chronic Obstructive Pulmonary Disease Using a Mobile Health System: Retrospective Analysis of a Randomized Controlled Trial
Source: JMIR Mhealth Uhealth. 2019 Nov 22;7(11):e14946. doi: 10.2196/14946 (PMC6898889; doi:10.2196/14946)
Supplement: Multimedia Appendix 1 [file mhealth_v7i11e14946_app1.docx]

# SUPPLEMENTARY MATERIAL

**Supplementary Table 1.** Overview of statistical comparisons for adherent participants compared with those non-adherent with using the system

|  | Adherent  (n=80) | Non-adherent (n=14) | P value |
| --- | --- | --- | --- |
| Age | 70.1 (9) | 67.6 (11) | 0.3647 |
| FEV_1_ | 48.7 (16.1) | 42.4 (14.5) | 0.3027 |
| Smoking pack-years - median(q1-q3) | 40 (29.5-58) | 30 (25-47) | 0.1073 |
| Smoking status | | | **<0.0001** |
| Current smoker, n(%) | 10 (12.5) | 8 (57.1) |  |
| Ex-smoker (<2 years), n(%) | 12 (15) | 1 (7.1) |  |
| Ex-smoker (≥2 years), n(%) | 58 (72.5) | 5 (35.7) |  |
| EQ-5D index - median(q1-q3) | 0.6 (0.5-0.7) | 0.6 (0.5-0.7) | 0.8620 |
| SGRQ-C | 56.2 (17.9) | 57.7 (24.3) | 0.7945 |
| SCL10 - median(q1-q3) | 2 (0-9) | 6 (1-9) | 0.2908 |
| SCL20a - median(q1-q3) | 10 (6.5-22) | 16 (8-27) | 0.4010 |
| BMQ | 32.6 (6.4) | 32.7 (4.8) | 0.9274 |
| MARS (non-para) | 24 (23-25) | 24 (24-25) | 0.2129 |
