# Supplementary material for: Mood Monitoring Over One Year for People With Chronic Obstructive Pulmonary Disease Using a Mobile Health System: Retrospective Analysis of a Randomized Controlled Trial
Source: JMIR Mhealth Uhealth. 2019 Nov 22;7(11):e14946. doi: 10.2196/14946 (PMC6898889; doi:10.2196/14946)
Supplement: Multimedia Appendix 2 [file mhealth_v7i11e14946_app2.docx]

# SUPPLEMENTARY MATERIAL

**Supplementary Table 2.** Overview of statistical comparisons for participants with elevated PHQ-8 and GAD-7 scores compared with those with non-elevated scores.

|  | Elevated PHQ-8 and GAD-7 scores  (n=47) | Non-elevated PHQ-8 and GAD-7 scores  (n=20) | P value |
| --- | --- | --- | --- |
| Age | 69.5 (9.6) | 70.7 (9) | 0.6528 |
| FEV_1_ | 46.5 (18.5) | 46.6 (14) | 0.9836 |
| Smoking pack-years - median(q1-q3) | 42 (30-53) | 40 (25-54.5) | 0.4927 |
| Smoking status | | | 0.989 |
| Current smoker, n(%) | 11 (23.4) | 5 (25) |  |
| Ex-smoker (<2 years), n(%) | 5 (10.6) | 2 (10) |  |
| Ex-smoker (≥2 years), n(%) | 31 (66) | 13 (65) |  |
| EQ-5D index - median(q1-q3) | 0.5 (0.4-0.7) | 0.7 (0.6-0.8) | **0.0078** |
| SGRQ-C | 63.3 (17.8) | 50.7 (16.8) | **0.0090** |
| SCL10 - median(q1-q3) | 6 (2-13) | 1 (0-4) | **0.0001** |
| SCL20a - median(q1-q3) | 22 (10-43) | 6 (3-9) | **<0.0001** |
| BMQ | 34.6 (5.6) | 32 (5.6) | 0.0838 |
| MARS - median(q1-q3) | 24 (22-24) | 22 (23.5-24) | 0.5164 |
